# Androgen signaling is dispensable for fetal testis formation: decoupling testis organogenesis from fetal masculinization programming

**DOI:** 10.1093/molehr/gaag044

**Published:** 2026-07-16

**Authors:** Caroline Despicht, Anna O Bindel, Anna K Rosenmai, Terje Svingen

**Affiliations:** National Food Institute, Technical University of Denmark, Kongens Lyngby, Denmark; National Food Institute, Technical University of Denmark, Kongens Lyngby, Denmark; National Food Institute, Technical University of Denmark, Kongens Lyngby, Denmark; National Food Institute, Technical University of Denmark, Kongens Lyngby, Denmark

**Keywords:** androgen receptor, AR, masculinization, androgen insensitivity syndrome, AIS, endocrine disruption

## Abstract

Androgen receptor (AR) signaling is central to male sexual differentiation. Notably, however, fetal testis formation and androgen-dependent masculinization are temporally and mechanistically distinct processes that are not always clearly differentiated in developmental and toxicological interpretations. Evidence from human androgen insensitivity syndromes (AIS) and rodent genetic models demonstrates fetal testis formation proceeds independently of AR, whereas downstream masculinization is AR-dependent. Across mammals, early testis differentiation is driven by intrinsic programs that establish Sertoli cells, organize testis cords, and specify fetal Leydig cells in the absence of AR activity. Consistently, mouse *Ar*-knockout models show persistent fetal testis formation, and individuals with complete androgen insensitivity syndrome (CAIS) develop structurally intact testes that produce normal or elevated testosterone despite absent virilization. These conserved outcomes reflect shared developmental timing, as key morphogenetic events occur before AR is expressed in Sertoli or fetal Leydig lineages in both rodents and humans. By contrast, AR-mediated androgen signaling becomes essential once androgens from the fetal testes begin to act on peripheral target tissues, governing masculinization of external genitalia, testis descent, and postnatal maturation of somatic testis compartments, as demonstrated by graded AIS phenotypes and cell-specific *Ar* ablation in mice. This developmental uncoupling explains why disruption of androgen signaling still allows establishment of early testis architecture yet predictably impairs downstream male reproductive development. Recognizing this distinction is crucial for interpreting human genetic disorders, aligning rodent models with human biology, but also to refine toxicological frameworks to target androgen-dependent windows of vulnerability.

## Introduction

In mammals, androgen signaling is central for establishing the male phenotype, guiding the transformation of an initially bipotential XY embryo into a masculinized organism (Wiener *et al.*, 1997; Matsumoto *et al.*, 2013). This process depends on the correct activation of the androgen receptor (AR) by its ligands testosterone and dihydrotestosterone (DHT) during a critical developmental interval often referred to as the masculinization programming window (Sharpe, 2020). Disruption of AR signaling during this period leads to degrees of under-virilization ranging from hypospadias or micropenis to complete feminization of the external genitalia in cases of severe androgen deficiency or non‑functional AR (Shukla *et al.*, 2016). The fetal testis is a main driver of this masculinization process through testosterone production that is transported to peripheral tissues. In tissues distal to the testes, testosterone can also be converted to the more potent DHT (Azzouni *et al.*, 2012; Delli Paoli *et al.*, 2023), which is required for directing male‑specific differentiation, including formation of the penis, scrotum, and perineal musculature.

In contrast, formation of the fetal testis follows a distinct developmental trajectory. Testis differentiation is initiated by the sex‑determining cascade, triggered by expression of SRY in pre‑Sertoli cells of the bipotential gonad. This leads to upregulation of SRY-box transcription factor 9 (SOX9) and subsequent specification of Sertoli cells, which organize into testis cords and orchestrate the differentiation of additional somatic lineages, including fetal Leydig cells (FLCs) (Wilhelm *et al.*, 2007; Sekido and Lovell-Badge, 2013). These early morphogenetic events occur before the onset of maximal androgen action and establish the cellular architecture required for later endocrine function. Consistent with this timing, FLCs initiate steroidogenesis after the establishment of testis cords, producing androgen precursors, with Sertoli cells being responsible for final testosterone synthesis in fetal testis (Shima *et al.*, 2013; O’Donnell *et al.*, 2022) that subsequently drive masculinization of peripheral tissues.

Despite these temporally distinct developmental processes, testis formation and androgen‑dependent masculinization are not always clearly separated when interpreting developmental, clinical, or experimental findings. Phenotypes such as under‑virilization are frequently used as indicators of disrupted male development more broadly, even though such outcomes may arise from altered androgen action due to impaired receptor function or reduced androgen synthesis, as seen for example in 17β‑hydroxysteroid dehydrogenase type 3 deficiency (Mendonca *et al.*, 2017), without necessarily reflecting defects in early gonadal formation. This interpretation is reinforced by the *AR* gene being located on the X chromosome, whereby loss-of-function mutations result in systemic androgen insensitivity despite preserved testis formation (Brown *et al.*, 1989). This distinction is particularly relevant in the context of human disorders of sex development and in experimental models where androgen signaling can be disrupted at different levels.

In this Review, we synthesize evidence from human clinical studies, including androgen insensitivity syndromes (AIS), together with data from global and cell‑specific *Ar* knockout (ARKO) models and comparative analyses of AR expression across species. Our aim is to delineate the temporally and functionally distinct roles of AR signaling during male reproductive development and to integrate these findings into a coherent developmental framework that distinguishes androgen‑independent testis organogenesis from androgen‑dependent masculinization and postnatal maturation.

## Methods

Relevant literature was identified through targeted searches of PubMed and Google Scholar and cross-referencing of key developmental biology and endocrine studies. Only human, mouse, and rat studies were included, as these species represent the most widely used models for human reproductive development and have the most comprehensive datasets on testis AR expression. Search terms such as ‘Androgen receptor expression’, ‘fetal’, ‘Leydig cells’, ‘Sertoli cells’, ‘peritubular myoid cells’, ‘germ cells’, ‘testicular cells’, ‘human’, ‘mouse’, ‘rat’, ‘ARKO’, ‘Tfm’, and ‘Androgen insensitivity syndrome’ were used individually or in combination. Studies were excluded if they did not include fetal developmental stages, or were conducted in species other than human, mouse, or rat. The cut-off date for the literature search was 22 April 2026. To map AR expression during fetal testis development, we compiled published studies reporting AR localization and expression levels in specific fetal testis cell types in humans and rodents. In most studies, spatiotemporal AR expression was assessed using immunohistochemistry (IHC); in one study, *AR* expression in isolated gonocytes was additionally confirmed by RT-PCR. Only studies reporting AR expression in human, mouse, or rat fetal testis were included. Beyond these species and developmental-stage restrictions, no formal quality assessment or exclusion criteria were applied. This approach was chosen for two reasons. First, the print quality and image resolution of some published IHC stainings were insufficient to allow for a reliable and objective reevaluation, and assessment of staining intensity was therefore left to the original authors’ interpretations. Second, we aimed to capture and illustrate discrepancies and variability across published literature, rather than to exclude divergent findings.

**Figure 1.**
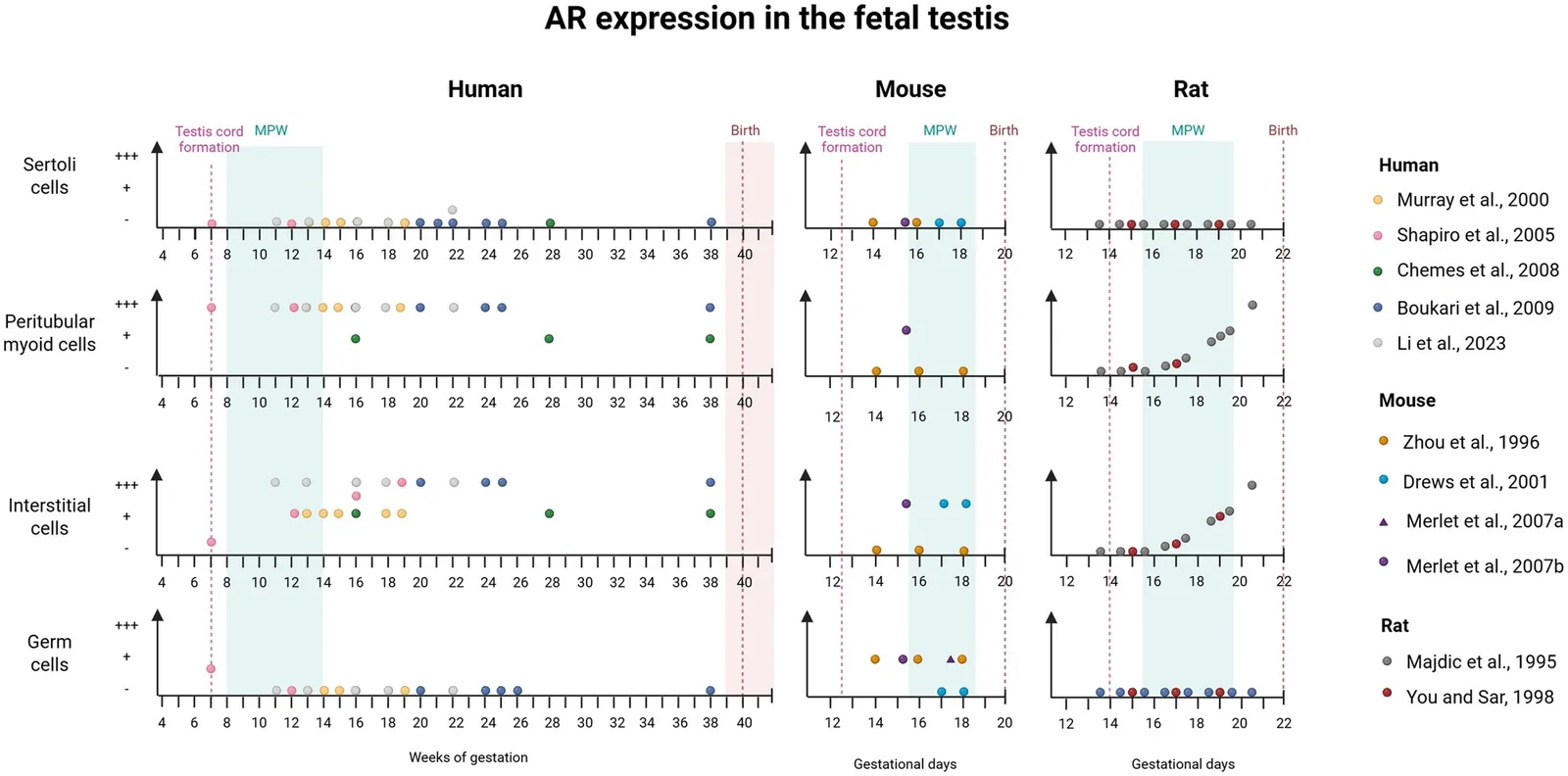
**Semi-quantitative overview of cell-type- and stage-specific androgen receptor (AR) expression**. AR expression in human and rodent testis cells, compiled from multiple literature sources (Majdic *et al*., 1995; Zhou *et al*., 1996; You and Sar, 1998; Murray *et al*., 2000; Drews *et al*., 2001; Shapiro *et al*., 2005; Merlet *et al*., 2007a,b; Chemes *et al*., 2008; Boukari *et al*., 2009; Li *et al*., 2023) Across all studies, AR expression was assessed by immunohistochemistry (IHC), depicted by circles (○) or by RT-PCR in micro dissected gonocytes, depicted by triangles (Δ). The *y*-axis indicates relative expression levels: (−) absent, (+) moderate, and (+++) strong., based on descriptions provided in the original publications. When data from multiple independent studies reported identical temporal and cellular AR expression patterns, corresponding data points overlap. In such cases, overlapping points are superimposed in the figure, and the color shown corresponds to the most recent study. Where authors reported temporal changes in expression intensity, these were reflected accordingly. When no change in intensity was described, data points were plotted at the same level across time. As the studies included did not quantitatively compare AR expression levels between cell types, across studies or between species, the relative placement of data points from different sources is a best-estimate depiction based on the available information. Accordingly, this figure is intended for illustrative purposes only. Created in BioRender. Despicht, C. (2026) https://BioRender.com/ex4yn1o MPW, Masculinization programming window.

For the preparation of Fig. 1, AR expression was broadly categorized into semi-quantitative levels ‘absent’ (no staining), ‘moderate’, or ‘strong’, based on the descriptions provided in the original publications. The figure was manually curated by classifying reported expression patterns into these defined categories and mapping them onto developmental timelines. Where AR expression intensity was not explicitly reported, expression was assigned to the intermediate category (moderate) for graphical representation. Where authors reported temporal changes in expression intensity, these were reflected accordingly. When no change in intensity was described, data points were plotted at the same level across time. As the included studies did not provide direct quantitative comparisons across cell types, studies, or species, the resulting visualization represents a best-estimate depiction of the available data and is intended for illustrative purposes. In addition to the IHC and RT-qPCR data included in Fig. 1, we examined publicly available human and mouse fetal testis single-cell RNA sequencing (scRNA-seq) datasets using the ReproGenomics Viewer (Darde *et al.*, 2019), to further assess *AR* expression patterns. Expression of *AR*/*Ar* was qualitatively evaluated across available developmental stages by visual inspection of gene expression patterns in UMAP representations of annotated testicular cell types. Only mouse and human scRNA-seq atlases were examined, as to our knowledge, no corresponding fetal rat testis dataset has been published to this date.

## Results

### Developmental timing of testis formation and androgen signaling

In contrast to masculinization of the peripheral reproductive system, which depends critically on AR‑mediated signaling during the masculinization programming window (Welsh *et al.*, 2008; Elmelund *et al.*, 2026), the fetal testis initiates differentiation largely without AR involvement, and the limited expression of AR across cell types in the fetal testis underscores this developmental division between fetal and adult stages. To illustrate this, Fig. 1 summarizes reported spatiotemporal AR expression timeline in major fetal testis cell types (Sertoli, peritubular, interstitial, and germ) in human, mouse, and rat.

In a majority of mammals, gonadal sex determination is initiated by the expression of the gene Sex determining region on the Y-chromosome (*SRY*) within the undifferentiated genital ridges of the XY embryo (Polanco and Koopman, 2007). In the mouse, *Sry* expression initiates around embryonic day (E)11.5 in the bipotential gonad, where it induces rapid upregulation of the transcription factor *Sox9*. The correct spatiotemporal expression of SOX9 drives the specification of Sertoli cells and their subsequent organization into testis cords, enclosing gonocytes that have previously migrated from adjacent tissues. Testis cord formation coincides with vascularization; following this, additional cell types emerge, with FLCs appearing in the interstitium around E12.5, and peritubular myoid cells (PMCs) surrounding testis cords around E13.5 (Brennan and Capel, 2004; Svingen and Koopman, 2013). This timeline of early testis formation is broadly conserved across species, with E11.5–12.5 in mice corresponding to approximately E13–14 in laboratory rats and gestational weeks (GWs) 6–7 in humans (Magre and Jost, 1980; Karl and Capel, 1995; Levine *et al.*, 2000; Guo *et al.*, 2021).

There is broad consensus that, in human testis, AR is absent from Sertoli cells until birth (Murray *et al.*, 2000; Shapiro *et al.*, 2005; Chemes *et al.*, 2008; Boukari *et al.*, 2009), underscoring once again that the aggregation of Sertoli cells into tubular structures must be occurring independently of androgen-signaling. Only one publication reported ‘intracordal staining’ from GW 20, which was attributed to Sertoli cells (Li *et al.*, 2023). Otherwise, the above findings are in accordance with the absence of AR in fetal Sertoli cells in mouse (Zhou *et al.*, 1996; Drews *et al.*, 2001; Merlet *et al.*, 2007b) and rat testes (Majdic *et al.*, 1995; You and Sar, 1998). In contrast, AR becomes highly relevant and expressed in Sertoli cells postnatally, where it is required for Sertoli cell maturation and androgen-regulated spermatogenesis in adulthood. Hence, it is thought that the fetal ‘androgen-insensitivity’ of Sertoli cells might serve to protect the fetal testis from precocious Sertoli (and thereby germ cell) maturation (Chemes *et al.*, 2008).

In contrast, AR appears to be present in PMCs and interstitial cells from early fetal stages. In human fetuses, AR is detected in PMCs as early as GW7 and continues to be highly expressed thereafter (Murray *et al.*, 2000; Shapiro *et al.*, 2005; Chemes *et al.*, 2008; Boukari *et al.*, 2009). This progressive increase in PMC AR-expression (occurring after testis formation) has also been documented in rat (Majdic *et al.*, 1995; You and Sar, 1998). Fetal PMCs contribute to the structural integrity of the testis by forming a circumferential layer around testis cords and expressing extracellular matrix components; however, the specific function of AR signaling in fetal PMCs is still unclear, as the most clear AR-mediated effect (i.e. tubular contractility, paracrine support spermatogenesis) are first observed postnatally (Jezek *et al.*, 1996; Merlet *et al.*, 2007a; Cowan *et al.*, 2010; Welsh *et al.*, 2012).

The term ‘interstitial’ encompasses Leydig cells, mesenchymal cells, as well as other cell types located between the testis cords, as immunohistochemical analyses generally do not distinguish between interstitial cell subtypes unless co‑staining with cell-specific markers is performed. In interstitial cells of the human fetal testis, AR is low at GW7 but increases through GW22 (Shapiro *et al.*, 2005), a gradual increase which also takes place from GD15 to GD21 in the developing rat testis. Interestingly, co-staining in the rat testis showed that 3β-hydroxysteroid dehydrogenase-positive cells (FLCs), were not the ones containing AR. Instead, it is a mesenchymal-like interstitial cell population that appeared AR positive, and the authors originally speculated these may be FLC progenitors dependent on androgen-signaling for their differentiation (Majdic *et al.*, 1995). However, it has since been shown that the AR-positive interstitial cells most likely serve as a progenitor pool for adult Leydig cells (ALCs) rather than FLCs (Kilcoyne *et al.*, 2014; Shima, 2019).

There is general consensus that AR is not expressed in (fetal) germ cells, as demonstrated by most studies presented in Fig. 1. However, a few mouse studies have reported that gonocytes do express AR and that androgens are required for gonocyte proliferation (Zhou *et al.*, 1996; Merlet *et al.*, 2007a,b). The functional role of AR in germ cells has since been further investigated using germ cell-specific ARKO models and will be discussed below. In humans, only one study reports weak AR staining in human undifferentiated gonadal cells in GW7, which is not detectable at later stages (GW12–GW22) (Shapiro *et al.*, 2005). As none of the studies reviewed here consistently assessed or compared AR expression across species, it remains unclear whether differences in AR expression and function in germ cells and other testis cell types reflect true species variation or instead arise from methodological differences, such as divergent IHC protocols or antibody specificity. To further address this, we examined *Ar* expression in available scRNA-seq datasets from mouse (Garcia-Alonso *et al.*, 2022; Mayère *et al.*, 2022) and human fetal testis (Guo *et al.*, 2021; Garcia-Alonso *et al.*, 2022; Lardenois *et al.*, 2026). Across human datasets, fetal *AR* expression is predominantly localized to gonadal mesenchymal cells, while expression in Leydig, Sertoli, and germ cells is either absent, or low and inconsistent. Unfortunately, PMCs were generally not annotated or represented in the UMAP visualizations. A similar pattern is observed in mouse atlases; however, in contrast to humans, *Ar* expression is also detected in fetal gonocytes shortly after testis formation (E13.5) (Mayère *et al.*, 2022).

To summarize, AR is absent or very low in Sertoli and FLCs from gonadal sex determination through to testis cord formation, consistent with their androgen-independent lineage commitment and contribution to testis formation. AR is detectable in a subset of PMCs and interstitial mesenchymal cells, potentially reflecting early involvement in extracellular matrix organization, stromal remodeling, and later lineage specification of ALCs, rather than primary sex determination. Although the overall pattern of AR expression is broadly conserved across species, timing and intensity of detected AR still seem to vary in those species where we have data, and it remains unclear whether AR might play slightly different roles in the fetal testis across species. Key knowledge gaps still remain regarding the fetal roles of AR-positive PMCs and interstitial cells, the onset of transcriptionally active AR across species, the influence of early AR activity on later Leydig maturation, and potential noncanonical AR functions in the fetal testis. Nevertheless, ARKO models in mice and phenotypic manifestations of human AIS have been central to improving our understanding of the cell- and developmental stage-specific functions of AR in the fetal testis and are therefore discussed in the following sections.

### Functional insights from ARKO mouse models

Mouse ARKO models provide direct experimental evidence for the developmental and cell type-specific roles of AR. Global and cell-specific ARKOs reveal a developmental transition from AR-independent fetal differentiation to AR-dependent postnatal function. Across various knockout models, a consistent pattern is seen, with fetal testis forming normally in the absence of *Ar*, whereas postnatal testis maturation and spermatogenesis depend critically on AR signaling (De Gendt and Verhoeven, 2012; O’Hara and Smith, 2015; Rey, 2021). Fetal and postnatal phenotypes of cell-specific and ubiquitous mouse ARKO models are summarized in Table 1.

**Table 1.** AR phenotypes of mouse knockout models and human mutation syndromes.

Mouse		
Model	Fetal/postnatal	Puberty/adulthood
Ubiquitous ARKO/Tfm	Testis forms but no descent; feminized genitalia; AR effects on gonocyte proliferation and PMC differentiation; SCs and FLCs lack AR (O’Shaughnessy *et al.*, 2002; De Gendt *et al.*, 2004; Tsai *et al.*, 2006; Merlet *et al.*, 2007a)	Testis present but small; pachytene arrest; azoospermia (De Gendt *et al.*, 2004; Tsai *et al.*, 2006)
SC-ARKO	Normal fetal development (De Gendt *et al.*, 2004; Tsai *et al.*, 2006)	Testis intact; reduced size; incomplete spermatogenesis (pachytene/diplotene) (De Gendt *et al.*, 2004; Tsai *et al.*, 2006)
LC-ARKO	FLCs unaffected; testis forms (Tsai *et al.*, 2006; O’Hara *et al.*, 2015)	ALC maturation defective; low T; GC arrest; infertility (Tsai *et al.*, 2006; O’Hara *et al.*, 2015)
GC-ARKO	No fetal abnormalities (Tsai *et al.*, 2006)	Testis near Wild type; spermatogenesis normal (Tsai *et al.*, 2006)
PMC-ARKO	Normal until puberty; indirect LC effects (Zhang *et al.*, 2006; Welsh *et al.*, 2008, 2009)	Disorganized epithelium; reduced GC; infertility (Zhang *et al.*, 2006; Welsh *et al.*, 2009)

Human		
Syndrome	AR mutations	Fetal/postnatal
CAIS	Various types across gene; enriched in ligand binding domain (Gottlieb *et al.*, 2012)	Testes formed; abdominal/inguinal; germ cells normal early then decline; female external genitalia (Hannema *et al.*, 2006; Mendonca *et al.*, 2009; Jääskeläinen, 2012)
PAIS	Single-base substitutions (Gottlieb *et al.*, 2012)	Testes formed; often undescended; variable external phenotype (Hannema *et al.*, 2006; Mendonca *et al.*, 2009; Jääskeläinen, 2012)
MAIS	Mild missense (Gottlieb *et al.*, 2012)	Testes intact; normal male genitalia (Jääskeläinen, 2012)
SBMA	CAG repeat expansion (Dejager *et al.*, 2002)	Normal testes; adult onset (Dejager *et al.*, 2002)

ARKO, androgen receptor knockout mouse; Tfm, testicular-feminized mutant mouse; SC-ARKO, Sertoli cell-ARKO mouse; LC-ARKO, Leydig cell-ARKO mouse; GC-ARKO, germ cell-ARKO mouse; PMC-ARKO, peritubular myoid cell-ARKO mouse; CAIS, complete androgen insufficiency syndrome; PAIS, partial androgen insufficiency syndrome; MAIS, mild androgen insufficiency syndrome; SBMA, spinal and bulbar muscular atrophy syndrome (*aka* Kennedy disease).

#### Global ARKO models

ARKO/Tfm (testicular-feminized) fetuses develop testes containing Sertoli and FLCs, yet internal and external genitalia are feminized, and testis descent fails, highlighting androgen-dependent processes in gubernacular and inguinoscrotal development (Zhou, 2010; Chang *et al.*, 2013). Despite structurally intact fetal testes, postnatal maturation is severely compromised, with reduced testis weight, meiotic arrest of germ cells, and azoospermia, reflecting the requirements of the AR in Sertoli cell maturation and spermatogenesis (O’Shaughnessy *et al.*, 2002; De Gendt *et al.*, 2004; Tsai *et al.*, 2006; Merlet *et al.*, 2007a).

#### Cell-specific ARKO models

Cell-specific knockouts have clarified how AR function varies by cell lineage. Fetal testis differentiation is preserved in Sertoli-cell ARKO (SC‑ARKO) mice, but AR absence postnatally reduces testis size and arrests meiosis (Chang *et al.*, 2004; De Gendt *et al.*, 2004; Verhoeven *et al.*, 2010). Similarly, FLC differentiation proceeds without *Ar* in Leydig-cell ARKO (LC‑ARKO) mice (Merlet *et al.*, 2007a), yet ALC function fails (Tsai *et al.*, 2006; Zhou, 2010; O’Hara *et al.*, 2015). *Ar* in germ cells is dispensable, as seen in germ-cell ARKO (GC‑ARKO) mice where spermatogenesis remains intact, indicating that effects of androgens are mediated via the somatic compartment. Finally, although among the first to express *Ar*, PMC‑ARKO mice display intact fetal testis morphology (Zhang *et al.*, 2006; Welsh *et al.*, 2009). Postnatally, however, *Ar* expression in PMCs is required for seminiferous tubule organization, Leydig cell maturation, and germ-cell maintenance (Welsh *et al.*, 2008, 2009; Zhou, 2010).

Collectively, these mouse genetic studies reveal that the roles of AR in the testis are cell-type-specific, stage-specific, and interdependent. Fetal testis differentiation can be sustained in the absence of AR, whereas postnatal testis function depends on coordinated AR activity across Sertoli cells, Leydig cells, PMCs, and interactions between somatic and germ cells, ensuring structural integrity, steroidogenesis, and spermatogenesis (De Gendt and Verhoeven, 2012).

### Human AR mutations and AIS

Human *AR* mutations provide a clinical mirror to mouse knockout models, reinforcing that AR is dispensable for fetal testis formation but essential for postnatal development and function. The spectrum of AIS—ranging from complete (CAIS) to partial (PAIS) to mild (MAIS), including spinal and bulbar muscular atrophy (SBMA)—illustrates how varying degrees and types of AR dysfunction manifest across the male reproductive system (Mendonca *et al.*, 2009; Gottlieb *et al.*, 2012).

In CAIS, individuals develop fully formed testes, which are typically abdominal or inguinal due to failed androgen-dependent descent. Germ-cell numbers initially appear normal but decline during childhood, while seminiferous tubules may remain age-appropriate before progressive degeneration. External genitalia, however, show a clear AR-dependence as they follow a female developmental trajectory closely paralleling global ARKO phenotypes (Gottlieb *et al.*, 2012; Zhang *et al.*, 2021).

PAIS illustrates a spectrum of partial AR dysfunction. Fetal testes form, but descent is often incomplete, Wolffian derivatives may be partially stabilized, and external genitalia range from clitoromegaly or posterior labial fusion to hypospadias or an under-virilized penis. Pubertal virilization is partial, gynecomastia is common, and fertility is variable. MAIS, which is usually caused by mild missense variants, presents with fully descended testes and male external genitalia, but with subtler postnatal androgen-dependent traits such as gynecomastia, sparse sexual hair, and variable spermatogenic impairment (Sultan *et al.*, 2001; Gottlieb *et al.*, 2012). Finally, SBMA, which is caused by a CAG-repeat expansion in the *AR* gene, affects adults; testis development and genital differentiation are normal, but reproductive and neuromuscular function decline over time, reflecting both impaired AR function and toxic gain-of-function due to accumulation of the polyglutamine‑expanded receptor protein (Dejager *et al.*, 2002; Giorgetti and Lieberman, 2016).

### Cross‑species synthesis: fetal testis form independently of AR signaling

**Figure 2.**
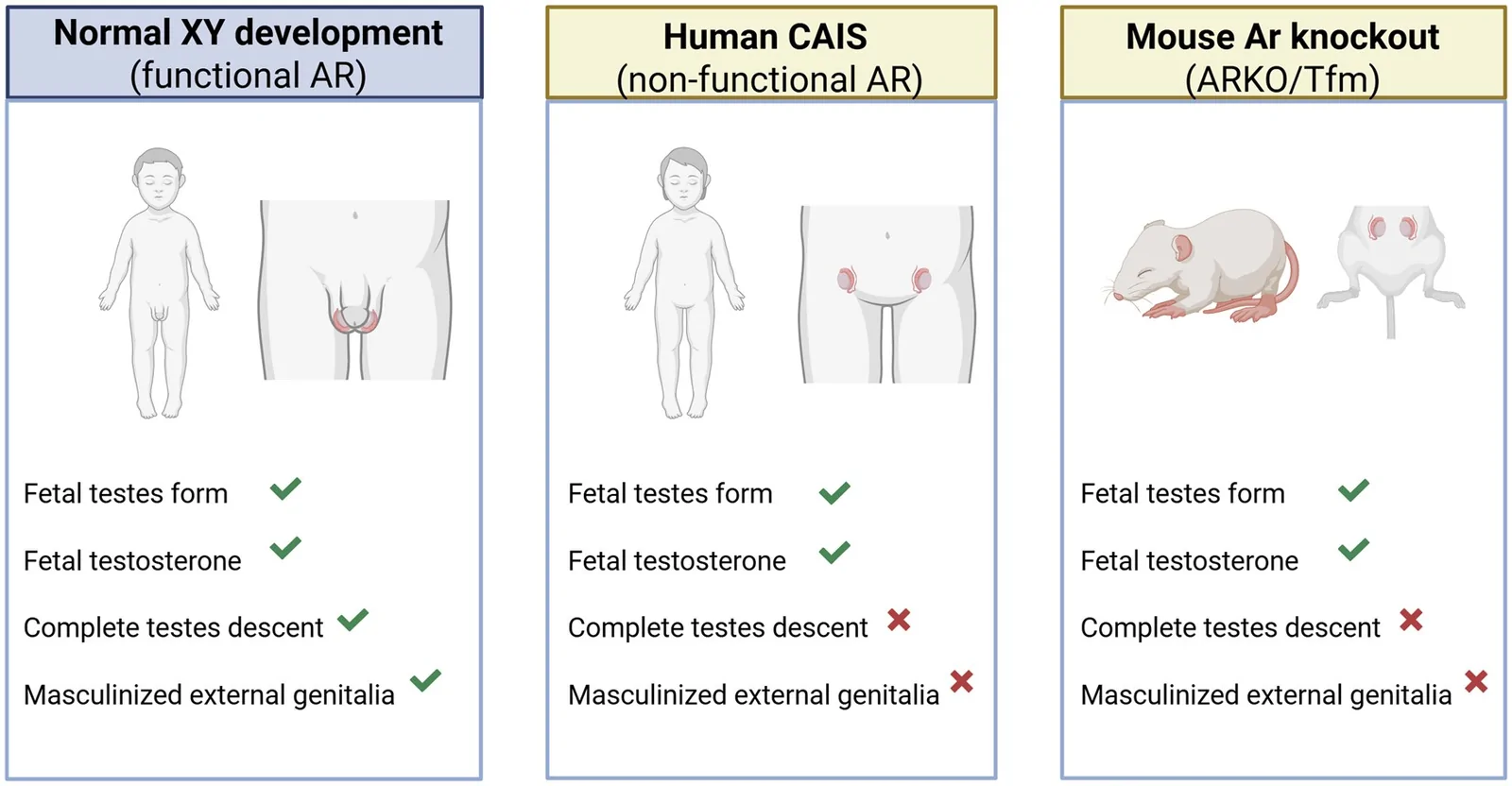
**Comparison of testis development and androgen‑dependent outcomes**. Fetal testes develop both in the presence and absence of functional androgen receptor (AR) signaling, but with secondary sex characteristics being affected when AR is non-functional. Created in BioRender. Despicht, C. (2026) https://BioRender.com/9kljgd8.

The integrating of clinical manifestation of human AIS with phenotypes of ARKO mouse models shows a conserved principle on male differentiation, where the testes form without AR influence, yet testis descent and postnatal function require AR (Fig. 2). This phenotypic evidence is further supported by the developmental timing of AR expression: both in rodents and humans, testis cords form before emergence of FLCs and PMCs and expression of AR in Sertoli cells, which only emerges late in gestation or postnatally. In adulthood, AR expression in Sertoli and Leydig cells is critical for postnatal germ-cell maintenance and spermatogenesis, while AR in PMCs and interstitial cells supports testis integrity.

Even though AR signaling is dispensable for building the fetal testis, it is essential for androgen-dependent sexual differentiation across multiple target tissues, including reproductive organs as well as other peripheral sites such as brain, muscle, and bone, and for processes such as testis descent and reproductive function in adulthood (Wang *et al.*, 2009; Walters *et al.*, 2010; Rana *et al.*, 2014). This is further exemplified by the two-phase process of testicular descent, where the transabdominal phase is regulated by INSL3 and proceeds largely independently of AR signaling, whereas the inguinoscrotal phase depends on androgen action (Klonisch *et al.*, 2004; Virtanen and Toppari, 2014).

## Perspectives and concluding remarks

Evidence from humans and rodents demonstrates that fetal testis differentiation occurs independently of AR signaling, with key morphogenetic events in across species preceding AR expression in Sertoli or FLCs. In contrast, AR-mediated androgen signaling is essential for early masculinization of external genitalia, testis descent, and in postnatal testis maturation. AR thus defines the functional output of the testis rather than its initial formation. This distinction explains why disruptions to androgen signaling can leave fetal testis morphology intact but produce predictable downstream consequences, from under-virilization in CAIS and PAIS to postnatal defects observed in cell-specific ARKO models.

This distinction has important implications for both clinical interpretation and experimental modeling. Decoupling AR-independent testis formation from AR-dependent masculinization can help refine the interpretation of disorders of sex development and improve cross-species alignment of model systems. Disruption of AR may spare testis formation while impairing androgen‑dependent extra‑testicular development, meaning that peripheral under-virilization is not necessarily predictive of gonadal dysgenesis. This highlights the importance of alternative, non-animal methods that capture tissue‑ and stage‑specific androgen‑dependent outcomes beyond testis morphology alone.

The same principle is highly relevant for reproductive toxicology. For instance, compounds that block AR or suppress fetal androgen production can leave testis morphology intact while impairing masculinization, descent, and postnatal maturation of Sertoli, Leydig, or PMCs, just as loss of AR function in animals and humans can lead to same phenotypic manifestations (Parks *et al.*, 2000; Fisher, 2004; Earl Gray Jr *et al.*, 2006). Consequently, fetal testis morphology alone is an insufficient endpoint for assessing androgen-dependent disruption.

These considerations underscore the need for more informative, tissue- and stage-specific endpoints, particularly in the development and interpretation of alternative and non-animal test methods. Assays that capture androgen-dependent outcomes beyond the testis will be essential for detecting biologically relevant perturbations of male reproductive development.

## Data Availability

No new data were generated or analyzed in support of this review.

## References

[gaag044-B1] Azzouni F , Godoy A, Li Y, Mohler J. The 5 alpha-reductase isozyme family: a review of basic biology and their role in human diseases. Adv Urol 2012;2012:530121. 22235201 10.1155/2012/530121 PMC3253436

[gaag044-B2] Boukari K , Meduri G, Brailly-Tabard S, Guibourdenche J, Ciampi ML, Massin N, Martinerie L, Picard J-Y, Rey R, Lombès M et al Lack of androgen receptor expression in Sertoli cells accounts for the absence of anti-Mullerian hormone repression during early human testis development. J Clin Endocrinol Metab 2009;94:1818–1825. 19276236 10.1210/jc.2008-1909 PMC2699416

[gaag044-B3] Brennan J , Capel B. One tissue, two fates: molecular genetic events that underlie testis versus ovary development. Nat Rev Genet 2004;5:509–521. 15211353 10.1038/nrg1381

[gaag044-B4] Brown CJ , Goss SJ, Lubahn DB, Joseph DR, Wilson EM, French FS, Willard HF. Androgen receptor locus on the human X chromosome: regional localization to Xq11-12 and description of a DNA polymorphism. Am J Hum Genet 1989;44:264–269. 2563196 PMC1715398

[gaag044-B5] Chang C , Chen Y-T, Yeh S-D, Xu Q, Wang R-S, Guillou F, Lardy H, Yeh S. Infertility with defective spermatogenesis and hypotestosteronemia in male mice lacking the androgen receptor in Sertoli cells. Proc Natl Acad Sci USA 2004;101:6876–6881. 15107499 10.1073/pnas.0307306101 PMC406435

[gaag044-B6] Chang C , Lee SO, Wang R-S, Yeh S, Chang T-M. Androgen receptor (AR) physiological roles in male and female reproductive systems: lessons learned from AR-knockout mice lacking AR in selective cells. Biol Reprod 2013;89:21. 23782840 10.1095/biolreprod.113.109132 PMC4076350

[gaag044-B7] Chemes HE , Rey RA, Nistal M, Regadera J, Musse M, González-Peramato P, Serrano A. Physiological androgen insensitivity of the fetal, neonatal, and early infantile testis is explained by the ontogeny of the androgen receptor expression in Sertoli cells. J Clin Endocrinol Metab 2008;93:4408–4412. 18713818 10.1210/jc.2008-0915

[gaag044-B8] Cowan G , Childs AJ, Anderson RA, Saunders PTK. Establishment of long-term monolayer cultures of somatic cells from human fetal testes and expansion of peritubular myoid cells in the presence of androgen. Reproduction 2010;139:749–757. 20089665 10.1530/REP-09-0532

[gaag044-B9] Darde TA , Lecluze E, Lardenois A, Stévant I, Alary N, Tüttelmann F, Collin O, Nef S, Jégou B, Rolland AD et al The ReproGenomics Viewer: a multi-omics and cross-species resource compatible with single-cell studies for the reproductive science community. Bioinformatics 2019;35:3133–3139. 30668675 10.1093/bioinformatics/btz047

[gaag044-B10] De Gendt K , Swinnen JV, Saunders PTK, Schoonjans L, Dewerchin M, Devos A, Tan K, Atanassova N, Claessens F, Lécureuil C et al A Sertoli cell-selective knockout of the androgen receptor causes spermatogenic arrest in meiosis. Proc Natl Acad Sci USA 2004;101:1327–1332. 14745012 10.1073/pnas.0308114100 PMC337052

[gaag044-B11] De Gendt K , Verhoeven G. Tissue- and cell-specific functions of the androgen receptor revealed through conditional knockout models in mice. Mol Cell Endocrinol 2012;352:13–25. 21871526 10.1016/j.mce.2011.08.008

[gaag044-B12] Dejager S , Bry-Gauillard H, Bruckert E, Eymard B, Salachas F, LeGuern E, Tardieu S, Chadarevian R, Giral P, Turpin G. A comprehensive endocrine description of Kennedy’s disease revealing androgen insensitivity linked to CAG repeat length. J Clin Endocrinol Metab 2002;87:3893–3901. 12161529 10.1210/jcem.87.8.8780

[gaag044-B13] Delli Paoli E , Di Chiano S, Paoli D, Lenzi A, Lombardo F, Pallotti F. Androgen insensitivity syndrome: a review. J Endocrinol Invest 2023;46:2237–2245. 37300628 10.1007/s40618-023-02127-y

[gaag044-B14] Drews U , Sulak O, Oppitz M. Immunohistochemical localisation of androgen receptor during sex-specific morphogenesis in the fetal mouse. Histochem Cell Biol 2001;116:427–439. 11735006 10.1007/s00418-001-0335-5

[gaag044-B15] Earl Gray L Jr , Wilson VS, Stoker T, Lambright C, Furr J, Noriega N, Howdeshell K, Ankley GT, Guillette L. Adverse effects of environmental antiandrogens and androgens on reproductive development in mammals. Int J Androl 2006;29:96–104. 16466529 10.1111/j.1365-2605.2005.00636.x

[gaag044-B16] Elmelund E , Draskau MK, Stewart MK, Mattiske DM, Pask AJ, Svingen T. Revisiting the dual role of androgens and oestrogens in mammalian sex differentiation with a focus on genitalia. Nat Rev Urol 2026;23:1–9. 41781713 10.1038/s41585-026-01132-z

[gaag044-B17] Fisher JS. Environmental anti-androgens and male reproductive health: focus on phthalates and testicular dysgenesis syndrome. Reproduction 2004;127:305–315. 15016950 10.1530/rep.1.00025

[gaag044-B18] Garcia-Alonso L , Lorenzi V, Mazzeo CI, Alves-Lopes JP, Roberts K, Sancho-Serra C, Engelbert J, Marečková M, Gruhn WH, Botting RA et al Single-cell roadmap of human gonadal development. Nature 2022;607:540–547. 35794482 10.1038/s41586-022-04918-4 PMC9300467

[gaag044-B19] Giorgetti E , Lieberman AP. Polyglutamine androgen receptor-mediated neuromuscular disease. Cell Mol Life Sci 2016;73:3991–3999. 27188284 10.1007/s00018-016-2275-1 PMC5045769

[gaag044-B20] Gottlieb B , Beitel LK, Nadarajah A, Paliouras M, Trifiro M. The androgen receptor gene mutations database: 2012 update. Hum Mutat 2012;33:887–894. 22334387 10.1002/humu.22046

[gaag044-B21] Guo J , Sosa E, Chitiashvili T, Nie X, Rojas EJ, Oliver E, Plath K, Hotaling JM, Stukenborg J-B, Clark AT et al; DonorConnect. Single-cell analysis of the developing human testis reveals somatic niche cell specification and fetal germline stem cell establishment. Cell Stem Cell 2021;28:764–778.e4. 33453151 10.1016/j.stem.2020.12.004 PMC8026516

[gaag044-B22] Hannema SE , Scott IS, Rajpert-De Meyts E, Skakkebaek NE, Coleman N, Hughes IA. Testicular development in the complete androgen insensitivity syndrome. J Pathol 2006;208:518–527. 16400621 10.1002/path.1890

[gaag044-B23] Jääskeläinen J. Molecular biology of androgen insensitivity. Mol Cell Endocrinol 2012;352:4–12. 21871529 10.1016/j.mce.2011.08.006

[gaag044-B24] Jezek D , Hittmair A, Rogatsch H, Kos M. Lamina propria of sex cords in human fetal testis: an immunohistological and stereological study. Anat Embryol (Berl) 1996;193:181–190. 8742059 10.1007/BF00214709

[gaag044-B25] Karl J , Capel B. Three-dimensional structure of the developing mouse genital ridge. Philos Trans R Soc Lond B Biol Sci 1995;350:235–242. 8570687 10.1098/rstb.1995.0157

[gaag044-B26] Kilcoyne KR , Smith LB, Atanassova N, Macpherson S, McKinnell C, Driesche S van den, Jobling MS, Chambers TJG, De Gendt K, Verhoeven G et al Fetal programming of adult Leydig cell function by androgenic effects on stem/progenitor cells. Proc Natl Acad Sci USA 2014;111:E1924–E1932. 24753613 10.1073/pnas.1320735111 PMC4020050

[gaag044-B27] Klonisch T , Fowler PA, Hombach-Klonisch S. Molecular and genetic regulation of testis descent and external genitalia development. Dev Biol 2004;270:1–18. 15136137 10.1016/j.ydbio.2004.02.018

[gaag044-B28] Lardenois A , Suglia A, Moore CL, Evrard B, Noël L, Rivaud P, Besson A, Toupin M, Blévinal J, Dumortier C et al Single-cell exploration of gonadal somatic cell lineage specification during human sex determination. Dev Cell 2026;61:400–415.e6. 41072414 10.1016/j.devcel.2025.09.011

[gaag044-B29] Levine E , Cupp AS, Skinner MK. Role of neurotropins in rat embryonic testis morphogenesis (cord formation)1. Biol Reprod 2000;62:132–142. 10611077 10.1095/biolreprod62.1.132

[gaag044-B30] Li Y , Overland M, Derpinghaus A, Aksel S, Cao M, Ladwig N, Cunha GR, Baskin LS. Development of the human fetal testis: morphology and expression of cellular differentiation markers. Differentiation 2023;129:17–36. 35490077 10.1016/j.diff.2022.03.002

[gaag044-B31] Magre S , Jost A. The initial phases of testicular organogenesis in the rat. An electron microscopy study. Arch Anat Microsc Morphol Exp 1980;69:297–318. 7212698

[gaag044-B32] Majdic G , Millar MR, Saunders PTK. Immunolocalisation of androgen receptor to interstitial cells in fetal rat testes and to mesenchymal and epithelial cells of associated ducts. J Endocrinol 1995;147:285–293. 7490558 10.1677/joe.0.1470285

[gaag044-B33] Matsumoto T , Sakari M, Okada M, Yokoyama A, Takahashi S, Kouzmenko A, Kato S. The androgen receptor in health and disease. Annu Rev Physiol 2013;75:201–224. 23157556 10.1146/annurev-physiol-030212-183656

[gaag044-B34] Mayère C , Regard V, Perea-Gomez A, Bunce C, Neirijnck Y, Djari C, Bellido-Carreras N, Sararols P, Reeves R, Greenaway S et al Origin, specification and differentiation of a rare supporting-like lineage in the developing mouse gonad. Sci Adv 2022;8:eabm0972. 35613264 10.1126/sciadv.abm0972 PMC10942771

[gaag044-B35] Mendonca BB , Domenice S, Arnhold IJP, Costa EMF. 46, XY disorders of sex development (DSD). Clin Endocrinol (Oxf) 2009;70:173–187. 18811725 10.1111/j.1365-2265.2008.03392.x

[gaag044-B36] Mendonca BB , Gomes NL, Costa EMF, Inacio M, Martin RM, Nishi MY, Carvalho FM, Tibor FD, Domenice S. 46, XY disorder of sex development (DSD) due to 17β-hydroxysteroid dehydrogenase type 3 deficiency. J Steroid Biochem Mol Biol 2017;165:79–85. 27163392 10.1016/j.jsbmb.2016.05.002

[gaag044-B37] Merlet J , Moreau E, Habert R, Racine C. Development of fetal testicular cells in androgen receptor deficient mice. Cell Cycle 2007a;6:2258–2262. 17890904 10.4161/cc.6.18.4654

[gaag044-B38] Merlet J , Racine C, Moreau E, Moreno SG, Habert R. Male fetal germ cells are targets for androgens that physiologically inhibit their proliferation. Proc Natl Acad Sci USA 2007b;104:3615–3620. 17360691 10.1073/pnas.0611421104 PMC1805536

[gaag044-B39] Murray TJ , Fowler PA, Abramovich DR, Haites N, Lea RG. Human fetal testis: second trimester proliferative and steroidogenic capacities1. J Clin Endocrinol Metab 2000;85:4812–4817. 11134148 10.1210/jcem.85.12.7046

[gaag044-B40] O’Donnell L , Whiley PAF, Loveland KL. Activin A and Sertoli cells: key to fetal testis steroidogenesis. Front Endocrinol 2022;13:898876. 10.3389/fendo.2022.898876 PMC9171382 35685219

[gaag044-B41] O’Hara L , McInnes K, Simitsidellis I, Morgan S, Atanassova N, Slowikowska-Hilczer J, Kula K, Szarras-Czapnik M, Milne L, Mitchell RT et al Autocrine androgen action is essential for Leydig cell maturation and function, and protects against late-onset Leydig cell apoptosis in both mice and men. FASEB J 2015;29:894–910. 25404712 10.1096/fj.14-255729 PMC4422361

[gaag044-B42] O’Hara L , Smith LB. Androgen receptor roles in spermatogenesis and infertility. Best Pract Res Clin Endocrinol Metab 2015;29:595–605. 26303086 10.1016/j.beem.2015.04.006

[gaag044-B43] O'Shaughnessy PJ , Johnston H, Willerton L, Baker PJ. Failure of normal adult Leydig cell development in androgen-receptor-deficient mice. J Cell Sci 2002;115:3491–3496. 12154079 10.1242/jcs.115.17.3491

[gaag044-B45] Parks LG , Ostby JS, Lambright CR, Abbott BD, Klinefelter GR, Barlow NJ, Gray LE Jr. The plasticizer diethylhexyl phthalate induces malformations by decreasing fetal testosterone synthesis during sexual differentiation in the male rat. Toxicol Sci 2000;58:339–349. 11099646 10.1093/toxsci/58.2.339

[gaag044-B46] Polanco JC , Koopman P. Sry and the hesitant beginnings of male development. Dev Biol 2007;302:13–24. 16996051 10.1016/j.ydbio.2006.08.049

[gaag044-B47] Rana K , Davey RA, Zajac JD. Human androgen deficiency: insights gained from androgen receptor knockout mouse models. Asian J Androl 2014;16:169–177. 24480924 10.4103/1008-682X.122590 PMC3955325

[gaag044-B48] Rey RA. The role of androgen signaling in male sexual development at puberty. Endocrinology 2021;162:bqaa215. 33211805 10.1210/endocr/bqaa215

[gaag044-B49] Sekido R , Lovell-Badge R. Genetic control of testis development. Sex Dev 2013;7:21–32. 22964823 10.1159/000342221

[gaag044-B50] Shapiro E , Huang H, Masch RJ, McFadden DE, Wu X-R, Ostrer H. Immunolocalization of androgen receptor and estrogen receptors α and β in human fetal testis and epididymis. J Urol 2005;174:1695–1698; discussion 1698. 16148684 10.1097/01.ju.0000179540.28209.de

[gaag044-B9268310] Sharpe RM. Androgens and the masculinization programming window: human-rodent differences. Biochem Soc Trans 2020;48:1725–1735. 32779695 10.1042/BST20200200 PMC7458408

[gaag044-B51] Shima Y. Development of fetal and adult Leydig cells. Reprod Med Biol 2019;18:323–330. 31607792 10.1002/rmb2.12287 PMC6780029

[gaag044-B52] Shima Y , Miyabayashi K, Haraguchi S, Arakawa T, Otake H, Baba T, Matsuzaki S, Shishido Y, Akiyama H, Tachibana T et al Contribution of Leydig and Sertoli cells to testosterone production in mouse fetal testes. Mol Endocrinol 2013;27:63–73. 23125070 10.1210/me.2012-1256 PMC5416943

[gaag044-B53] Shukla GC , Plaga AR, Shankar E, Gupta S. Androgen receptor-related diseases: what do we know? Andrology 2016;4:366–381. 26991422 10.1111/andr.12167

[gaag044-B54] Sultan C , Paris F, Terouanne B, Balaguer P, Georget V, Poujol N, Jeandel C, Lumbroso S, Nicolas JC. Disorders linked to insufficient androgen action in male children. Hum Reprod Update 2001;7:314–322. 11392378 10.1093/humupd/7.3.314

[gaag044-B55] Svingen T , Koopman P. Building the mammalian testis: origins, differentiation, and assembly of the component cell populations. Genes Dev 2013;27:2409–2426. 24240231 10.1101/gad.228080.113 PMC3841730

[gaag044-B56] Tsai M-Y , Yeh S-D, Wang R-S, Yeh S, Zhang C, Lin H-Y, Tzeng C-R, Chang C. Differential effects of spermatogenesis and fertility in mice lacking androgen receptor in individual testis cells. Proc Natl Acad Sci USA 2006;103:18975–18980. 17142319 10.1073/pnas.0608565103 PMC1748162

[gaag044-B57] Verhoeven G , Willems A, Denolet E, Swinnen JV, De Gendt K. Androgens and spermatogenesis: lessons from transgenic mouse models. Philos Trans R Soc Lond B Biol Sci 2010;365:1537–1556. 20403868 10.1098/rstb.2009.0117 PMC2871915

[gaag044-B58] Virtanen HE , Toppari J. Embryology and physiology of testicular development and descent. Pediatr Endocrinol Rev 2014;11:206–213. 24683945

[gaag044-B59] Walters KA , Simanainen U, Handelsman DJ. Molecular insights into androgen actions in male and female reproductive function from androgen receptor knockout models. Hum Reprod Update 2010;16:543–558. 20231167 10.1093/humupd/dmq003

[gaag044-B60] Wang R-S , Yeh S, Tzeng C-R, Chang C. Androgen receptor roles in spermatogenesis and fertility: lessons from testicular cell-specific androgen receptor knockout mice. Endocr Rev 2009;30:119–132. 19176467 10.1210/er.2008-0025 PMC2662628

[gaag044-B61] Welsh M , Moffat L, Belling K, De França LR, Segatelli TM, Saunders PTK, Sharpe RM, Smith LB. Androgen receptor signalling in peritubular myoid cells is essential for normal differentiation and function of adult Leydig cells. Int J Androl 2012;35:25–40. 21651570 10.1111/j.1365-2605.2011.01150.x

[gaag044-B62] Welsh M , Saunders PTK, Atanassova N, Sharpe RM, Smith LB. Androgen action via testicular peritubular myoid cells is essential for male fertility. FASEB J 2009;23:4218–4230. 19692648 10.1096/fj.09-138347 PMC2812048

[gaag044-B63] Welsh M , Saunders PTK, Fisken M, Scott HM, Hutchison GR, Smith LB, Sharpe RM. Identification in rats of a programming window for reproductive tract masculinization, disruption of which leads to hypospadias and cryptorchidism. J Clin Invest 2008;118:1479–1490. 18340380 10.1172/JCI34241 PMC2267017

[gaag044-B64] Wiener JS , Teague JL, Roth DR, Gonzales ET, Lamb DJ. Molecular biology and function of the androgen receptor in genital development. J Urol 1997;157:1377–1386. 9120959

[gaag044-B65] Wilhelm D , Palmer S, Koopman P. Sex determination and gonadal development in mammals. Physiol Rev 2007;87:1–28. 17237341 10.1152/physrev.00009.2006

[gaag044-B67] You L , Sar M. Androgen receptor expression in the testes and epididymides of prenatal and postnatal Sprague-Dawley rats. Endocr 1998;9:253–261. 10.1385/ENDO:9:3:253 10221590

[gaag044-B68] Zhang C , Yeh S, Chen Y-T, Wu C-C, Chuang K-H, Lin H-Y, Wang R-S, Chang Y-J, Mendis-Handagama C, Hu L et al Oligozoospermia with normal fertility in male mice lacking the androgen receptor in testis peritubular myoid cells. Proc Natl Acad Sci USA 2006;103:17718–17723. 17095600 10.1073/pnas.0608556103 PMC1693813

[gaag044-B69] Zhang D , Yao F, Tian T, Deng S, Luo M, Tian Q. Clinical characteristics and molecular genetics of complete androgen insensitivity syndrome patients: a series study of 30 cases from a Chinese tertiary medical center. Fertil Steril 2021;115:1270–1279. 33602557 10.1016/j.fertnstert.2020.12.008

[gaag044-B70] Zhou X. Roles of androgen receptor in male and female reproduction: lessons from global and cell-specific androgen receptor knockout (ARKO) mice. J Androl 2010;31:235–243. 19959826 10.2164/jandrol.109.009266

[gaag044-B71] Zhou X , Kudo A, Kawakami H, Hirano H. Immunohistochemical localization of androgen receptor in mouse testicular germ cells during fetal and postnatal development. Anat Rec 1996;245:509–518. 8800409 10.1002/(SICI)1097-0185(199607)245:3<509::AID-AR7>3.0.CO;2-M

